# Response of oxidative stress and inflammatory biomarkers to a 12-week aerobic exercise training in women with metabolic syndrome

**DOI:** 10.1186/s40798-015-0011-2

**Published:** 2015-04-08

**Authors:** Juliano Boufleur Farinha, Flávia Mariel Steckling, Sílvio Terra Stefanello, Manuela Sangoi Cardoso, Larissa Santos Nunes, Rômulo Pillon Barcelos, Thiago Duarte, Nélson Alexandre Kretzmann, Carlos Bolli Mota, Guilherme Bresciani, Rafael Noal Moresco, Marta Maria Medeiros Frescura Duarte, Daniela Lopes dos Santos, Félix Alexandre Antunes Soares

**Affiliations:** 1 Departamento de Métodos e Técnicas Desportivas, Universidade Federal de Santa Maria, Avenida Roraima 1000, Santa Maria, 97105-900 Brazil; 2 Escola Superior de Educação Física, Universidade Federal do Rio Grande do Sul, Rua Felizardo 750, Porto Alegre, 90690200 Brazil; 3 Departamento de Bioquímica e Biologia Molecular, Universidade Federal de Santa Maria, Avenida Roraima 1000, Santa Maria, 97105-900 Brazil; 4 Departamento de Análises Clínicas e Toxicológicas, Universidade Federal de Santa Maria, Avenida Roraima 1000, Santa Maria, 97105-900 Brazil; 5 Centro Universitário Franciscano, Rua dos Andradas 1614, Santa Maria, 97010-032 Brazil; 6 Hospital de Clínicas de Porto Alegre, Rua Ramiro Barcelos 2350, Porto Alegre, 90035-903 Brazil; 7 Facultad de Ciencias de la Salud, Universidade Autónoma de Chile, Avenida Alemania 01090, Temuco, 4810101 Chile; 8 Universidade Luterana do Brasil, BR 287 km 252, Santa Maria, 97020-001 Brazil

## Abstract

**Background:**

Evidences have been highlighted the relationship among metabolic syndrome, chronic low-grade inflammation, oxidative stress and several diseases. In this sense, the aim of this study was to investigate the effects of aerobic exercise training on oxidative stress and inflammatory parameters on women with metabolic syndrome (MS).

**Methods:**

Twenty-three untrained women (51.86 ± 6.58 years old, BMI 30.8 ± 4.3 kg/m^2^) completed a 12-week treadmill exercise training, without modifications on dietary pattern. Advanced oxidation protein products (AOPP), thiobarbituric acid-reactive substances (TBARS), total thiol content (T-SH) and nitrite and nitrate (NOx) levels were assessed in plasma while the levels of interleukin-1 beta (IL-1β), interleukin-6 (IL-6), interleukin-10 (IL-10), tumor necrosis factor alpha (TNF-α) and interferon-gamma (IFN-γ) were evaluated in the serum. The RNA expression (mRNA) of IL-1β, IL-10, TNF-α, IFN-γ, insulin receptor substrate 2 (IRS-2) and matrix metalloproteinase-9 (MMP-9) were performed inperipheral blood mononuclear cells (PBMC) of a subset with eight women with MS using real real-time polymerase chain reaction (qPCR).

**Results:**

The intervention resulted in decreased serum levels of IL-1β, IL-6, TNF-α, IFN-γ, AOPP and TBARS, besides increased levels of IL-10 and T-SH (*P* < 0.001). NOx concentrations were unchanged, similarly to mRNA expressions quantified in PBMC.

**Conclusions:**

Twelve weeks of AT improved systemic oxidative stress and inflammatory biomarkers in women with MS, although PBMC mRNA expression for inflammatory pathways appeared to be unchanged. This may indicate that AT induced beneficial effects not only in physical fitness but also on health promotion through decreased oxidative damage and proinflammatory status.

## Key points

- Moderate aerobic training improves serum/plasma oxidative stress and inflammatory biomarkers in women with metabolic syndrome, although mRNA expression in lymphocytes/monocytes does not change.
- One speculates that major weight loss afforded by exercise training is necessary to alter inflammatory gene expression levels in lymphocytes/monocytes, unlike inflammatory serum markers.

## Background

The metabolic syndrome (MS) is characterized by interrelated risk factors such as impaired glucose tolerance, hypertriglyceridemia, low high-density lipoprotein (HDL) levels, raised blood pressure, and obesity (particularly visceral adiposity) [1]. During the last years, MS has consistently increased worldwide, becoming a public health concern and a clinical condition highly related to increasing obesity incidence, sedentary lifestyle, and excessive caloric intake [1].

Additionally, it has been shown that MS and obesity are linked with chronic low-grade systemic inflammation, particularly higher in women [2,3]. In this regard, persistent low-grade inflammation is described by a considerable increase in the systemic levels of cytokines, such as tumor necrosis factor alpha (TNF-α), interleukin-1 beta (IL-1β), and interleukin-6 (IL-6), which presence is highly related to atherosclerosis, non-alcoholic fatty liver disease, and type 2 diabetes mellitus (T2DM) [4-6]. The increased cytokine levels facilitate the intracellular influx of peripheral blood mononuclear cells (PBMC), which include lymphocytes and monocytes, constituting an important component of the immune system. In this sense, a synergistic relationship between low-grade systemic inflammation and oxidative stress has also been postulated. Regarding this, cytokines and immune cells are able to trigger the production of reactive oxygen (ROS) and nitrogen species (RNS) to cope with defense activities [3]. On the opposite, unbalanced cytokine release results in increased ROS production and oxidative stress-related conditions [3], such as atherosclerosis, stroke, renal and liver disorders, rheumatoid arthritis, auto-immune deficiencies, cancer, and Alzheimer's and Parkinson's diseases [7,8]. Therefore, evidence point out to an interaction among low-grade systemic inflammation with ROS overproduction, leading to these oxidative stress- and inflammation-related conditions previously mentioned.

It has been recently stated that aerobic exercise training (AT) may be considered as the most effective non-pharmacological tool for MS treatment [9]. However, despite several studies which demonstrated the antioxidant and anti-inflammatory effects of AT [6,10,11], other trials do not present inflammatory and oxidative profiles after a short-duration moderate-intensity AT in T2DM and obese women [3,12]. Furthermore, studies involving messenger RNA (mRNA) inflammatory marker gene expression in PBMC have so far evaluated the acute effects of exercise or the outcomes of a diet program [13-16], while the consequences of an AT have not been reported yet in this population. Therefore, the aim of this study was to investigate the effects of a 12-week moderate-intensity aerobic training on inflammatory and oxidative stress parameters in middle-aged women with metabolic syndrome.

## Methods

### Participants

Through newspapers and institutional website advertisements, 30 untrained middle-aged women volunteered to participate in the study. Participants were selected based upon the following criteria: 1) at least two MS characteristics using the South American waist circumference cutpoints [1], 2) age between 40 and 64 years, and 3) a full medical screening with a sports science physician. Moreover, before the study onset, subjects were asked to not alter diet intake throughout the training schedule. The study was approved by the Ethics Committee of the Universidade Federal de Santa Maria (0032.0.243.000-07) and followed the statements of the Declaration of Helsinki. All participants gave their informed consent after being fully informed about experimental procedures.

### Study design

At baseline and after 12 weeks of AT, participants underwent a series of anthropometric measurements, cardiorespiratory fitness test, and resting heart rate (RHR) in order to assess anthropometric and functional profiles. Serum, plasma, and PBMC were used to evaluate biochemical, oxidative stress, and inflammatory status. To minimize a possible nutritional bias, participants were encouraged to maintain the habitual dietary intake during the study and filled in a 3-day diet record. During the sessions of exercise schedule, training intensity was controlled with heart rate monitors.

### Training protocol

The AT was designed according to the recommendations of physical training for MS patients [17] (Table 1). The protocol consisted on treadmill brisk walking and/or slow jogging 3 days per week (48-h rest between sessions). In each day, the subjects were divided in six groups (three or four volunteers per class), where the patients were accompanied by two or three physical trainers per class, thus, affording almost a personal treatment for intensity accompaniment and constant motivation. We applied overloads in duration and/or intensity every 1 or 2 weeks to promote several adaptations, taking into consideration that the patients were previously untrained and presented MS. The large variability of sessions' duration and intensity are explained by the need of gradual progression of volume and intensity loads [17]. Taking into account the risk of cardiovascular complications in middle-aged and older adults, this risk can be minimized by beginning a training schedule at light-to-moderate intensity, employing a gradual progression of the volume of the physical training, as stated by the principles of training [17]. The intensity was controlled across the target heart rate reserve (HRR) [18]. The training sessions started with a 5-min warm-up performed on treadmills at approximately 40% HRR and ended up with a 5-min recovery and stretching. All training sessions were individually supervised, and the heart rate was continuously monitored using heart rate monitors (Polar RS 400, Polar Electro Oy, Kempele, Finland). The AT duration and intensity of the sessions ranged from 30 to 60 min and 50 to 65% of the HRR [17] during the 12 weeks of the training schedule.

**Table 1:** **Aerobic training schedule**

**Week**	**Duration (min)**	**Intensity (% of HRR)**
1	30	50
2	35	55
3-4	40	55
5	40	60
6-7	45	60
8	45	65
9-10	50	65
11	55	70
12	60	70

HRR, heart rate reserve.

### Resting heart rate, blood pressure, and cardiorespiratory fitness assessment

The subject's RHR was measured during 5 min in supine position, and the lower value was registered by a heart rate monitor (Polar RS 400, Polar Electro Oy, Kempele, Finland). After, resting systolic and diastolic pressures were measured after participants sat quietly in a padded chair for 5 min with a digital sphygmomanometer (Omron, Kyoto, Japan). The cardiorespiratory fitness was assessed by a graded treadmill test (ATL-10.100, Inbramed, Porto Alegre, Brazil) according to the Bruce's modified protocol [19], and the maximal oxygen uptake (VO_2_max) was calculated as previously described [17]. Participants were verbally encouraged to perform the maximum effort during the test. In this submaximal test, the VO_2_max was achieved by attaining heart rate over 85% of age-predicted maximum and volitional fatigue according to previous recommendations [17].

### Anthropometry

Subjects were weighed with a scale (Plenna, São Paulo, Brazil) and height measured with a stadiometer (Cardiomed, Curitiba, Brazil). The waist-hip ratio was determined by measuring waist circumference at the narrowest region between the costal margin and iliac crest and dividing by the hip circumference measured at its greatest gluteal protuberance by a spring-loaded metal tape. The body lean mass, body fat mass, trunk fat mass, arm fat mass, and leg fat mass were determined using dual-energy X-ray absorptiometry (DXA) with a densitometer machine (Hologic Discovery QDR, Bedford, USA). Briefly, after a 12-h fasting and 24 h without exercising, participants wore only a light coat, lay on the DXA table in supine position with arms adequately separated from the trunk and were asked to remain still throughout the scanning procedure.

### Blood collection and preparation

Blood samples were drawn from the vein of the antecubital region following a 12-h fasting, and participants were asked to avoid intense physical exercise 72 h before sampling. All blood samples were drawn into 4-mL EDTA anticoagulant or serum separator tubes. Serum and plasma were routinely centrifuged at 1,500×*g* for 15 min. Plasma and serum were stored at −80°C for subsequent analysis. PBMC were immediately separated from anticoagulated peripheral blood by density gradient centrifugation with Histopaque®-1077 solution (Sigma-Aldrich, St. Louis, USA), as previously described [16],and with short modifications. For each sample, four 15-mL centrifuge tubes were used to layer 7 mL of blood and phosphate-buffered saline (PBS) (136 mM NaCl, 2.7 mM KCl, 7.8 mM Na_2_HPO_4_, 1.7 mM KH_2_PO_4_) onto 3 mL of Histopaque®-1077. The suspension was centrifuged for 40 min at 275×*g* at room temperature. After, the mononuclear cell layer was removed with manual pipetting and washed in PBS. Then, cell supernatants were discarded, and the PBMC pellets were dried out with lysing solution (150 mM NH_4_Cl, 10 mM NaHCO_3_, 1 mM EDTA) and centrifuged for 3 min at 300×*g*. Finally, samples were stored with RNAlater® (Sigma-Aldrich, St. Louis, USA) and frozen at −80°C for further analysis.

### Serum biochemical assays

Total cholesterol and HDL were determined using commercially available assay kits (Bioclin, Belo Horizonte, Brazil) on a Cobas MIRA® automated analyzer (Roche Diagnostics, Basel, Switzerland). Serum triglycerides (TG) and glucose were determined using commercial kits (Bio Técnica, Varginha, Brazil). The cytokine levels were determined by enzyme-linked immunosorbent assay (ELISA) using commercial kits for human IL-1β, IL-6, interleukin-10 (IL-10), TNF-α, and interferon-gamma (IFN-γ), according to manufacturer's instructions (eBIOSCIENCE, San Diego, USA). The IL-1β, IL-6, and IL-10 kits were sensitive to 2 pg/mL. TNF-α and IFN-γ kits presented sensitivities of 4 pg/mL and 4 μg/mL, respectively.

### Plasma assays

Oxidative damage to proteins was measured by advanced oxidation protein products (AOPP) as previously described [20]. Shortly, plasma and chloramine-T calibration standards were transferred to test tubes. Potassium iodide (KI) (Sigma-Aldrich, St. Louis, USA) was added to the standards, and citric acid was added to the plasma samples. After 2 min on a tube shaker, the absorbance was read at 340 nm against a solvent blank (citric acid and KI). AOPP concentrations are expressed as micromole per liter of chloramine-T equivalents, abbreviated to μmol/L in this article. As an index of lipoperoxidation, thiobarbituric acid-reactive substances (TBARS) were assayed as previously described [21]. TBARS consists in an acid-heating reaction of the lipid peroxidation end product, malondialdehyde (MDA), with thiobarbituric acid (TBA). TBARS were determined spectrophotometrically at 532 nm and expressed as microgram per deciliter of MDA.

Nitrite and nitrate (NOx) levels were assessed by a modified Griess method. Briefly, samples were pippeted into the reaction cuvette, and vanadium (III) chloride (VCl_3_) (Sigma-Aldrich, St. Louis, USA) was added to reduce nitrate to nitrite after 25 s. Thus, Griess reagent (Sigma-Aldrich, St. Louis, USA) was added, and the resultant mixture was incubated for 20 min and read at 550 nm [22]. Creatinine content was determined using a commercially available assay kit (Labtest, Lagoa Santa, Brazil). Plasma levels of AOPP, creatinine, and NOx were measured on an automated analyzer Cobas MIRA® (Roche Diagnostics, Basel, Switzerland). Moreover, total thiol group concentrations (T-SH) were assessed by reacting with [5,5′-dithiobis(2-nitrobenzoic acid); DTNB] [23], read at 412 nm, and expressed in nanomole per milligram of glutathione (GSH).

### PBMC gene mRNA expression

Total RNA was isolated from PBMC using RiboPure™ Blood Kit (Ambion Inc., Austin, USA) and quantified by the fluorescent method Ribogreen RNA Quantification Kit (Molecular Probes, Leiden, The Netherlands), as previously described [16]. The integrity of the RNA was assessed using agarose gel electrophoresis. RNA was reverse-transcribed using random hexamer primers. Real-time polymerase chain reaction (qPCR) was performed using SYBR Green I reagent (Applied Biosystems, Foster City, USA) with Mx3000P real-time PCR Stratagene (GE) system. Data was processed by the fully integrated MX PRO software. Relative changes in gene expression levels were determined using the 2^−ΔΔct^ method as described previously [24]. Shortly, this method requires the assignment of one housekeeping gene (β-actin in this case), which is assumed to be constantly expressed in all samples. Then, the expression of other samples is compared to that in reference sample. TaqMan primers and probes for IL-1β, IL-10, TNF-α, IFN-γ, insulin receptor substrate 2 (IRS-2), matrix metalloproteinase-9 (MMP-9), and β-actin (Table 2) were designed from the commercially available TaqMan® Assays-on-Demand Gene (Invitrogen, Carlsbad, USA). Considering the dual role of interleukin-6 (IL-6) as a proinflammatory and sometimes anti-inflammatory (pleiotropic effects) mediator according to the tissue (cell-specific actions) [6,14], we have decided to rule it out and focus on well-established proinflammatory or anti-inflammatory markers. Regarding questions concerning available funds, the researchers analyzed the patients which more responded to the training (higher deltas) in relation to serum cytokine levels and utilized the PBMC samples previously stored for these eight patients to perform qPCR assays. Measurements were performed at baseline and at the end of the study, with samples analyzed in duplicate.

**Table 2:** **Primers used in qPCR**

**Gene**	**Sense primers (5′→3′)**	**Anti-sense primers (5′→3′)**	**Fragment length (bp)**	**Annealing temperature (°C)**	**Mean Ct BT**	**Mean Ct AT**
IL-1β	ATGATGGCTTATTACAGTGGCAA	GTCGGAGATTCGTAGCTGGA	132	57	38.9	37.7
IL-10	GACTTTAAGGGTTACCTGGGTTG	TCACATGCGCCTTGATGTCG	112	58	34.1	36.6
TNF-α	GAGGCCAAGCCCTGGTATG	CGGGCCGATTGATCTCAGC	91	57	37.6	37.7
IFN-γ	TCGGTAACTGACTTGAATGTCCA	TCGCTTCCCTGTTTTAGCTGC	93	57	31.9	34
IRS-2	CCCGACTTCTTCTCCGCAG	GAAGGCACTACAGGGTGAGG	124	58	35.1	35.3
MMP-9	TGGGCTACGTGACCTATGACAT	GCCCAGCCCACCTCCACTCCTC	128	54	32.2	34.9
β-actin	GCTTCTTTGCAGCTCCTTCGT	ATATCGTCATCCATGGCGAAC	150	56	32.6	35.6

Ct, threshold cycle; BT, before training; AT, after training.

### Dietary intake assessment

In order to determine total daily caloric and macronutrient intake, participants were instructed to self-report the exact amount of food and drink consumed over two weekdays and one weekend day in a 3-day diet record. Energy intake was determined using a dietary analysis software (Avanutri Revolution, Rio de Janeiro, Brazil).

### Statistical analysis

The Shapiro-Wilk test was carried out to assess the normality of variable distribution. According the normality of data, paired Student's *t* test and Wilcoxon signed rank test were utilized to determine differences pre and post training. PBMC parameters were compared by Student's *t* test for independent samples or by the Mann-Whitney U test. Statistical Package for Social Sciences (SPSS 15.0, Chicago, USA) was used, and statistical significance set at *P* < 0.05. Data were expressed as mean ± standard deviation of mean (SD).

## Results

### Sample characterization

From the initial sample, 23 participants (18 postmenopausal and 5 participants with regular menses) aging 51.86 ± 6.58 years old and presenting 158 cm of height completed the training schedule and were considered in the statistical analysis. There were no significant differences between postmenopausal women and those with regular menses on biochemical, oxidative stress, and inflammatory blood parameters before and/or after AT (data not shown). None of the participants used any form of hormone replacement therapy. From the initial sample, three patients dropped out due to health problems unrelated to the study and five for private reasons. Overall, 82.61% (*n* = 19) of the participants presented three or more MS characteristics at baseline, and the adherence to the exercise training was 84.65% (number of sessions attended × 100/number of sessions offered) throughout the study.

### Changes in anthropometric, functional, and biochemical serum parameters

The AT did not alter the hip circumference, RHR, and systolic and diastolic blood pressure values. However, the participants exhibited a significant decrease in body weight (*P* < 0.001), body mass index (BMI) (*P* < 0.001), waist circumference (*P* < 0.001), waist-hip ratio (*P* = 0.004), total body fat mass (*P* < 0.001), truncal fat mass (*P* < 0.001), arm fat mass (*P* = 0.015), leg fat mass (*P* < 0.001) and an increase in total body lean mass (*P* = 0.03) (Table 3). With regard to cardiorespiratory fitness, participants showed a greater VO_2_max levels (*P* < 0.001) and a longer time spent in the test (*P* < 0.001) after the training. Although glucose, total cholesterol, TG, and HDL levels were unchanged, creatinine concentration decreased after the intervention (*P* = 0.017) (Table 3).

**Table 3:** **Anthropometric, biochemical, and functional characteristics of the participants before and after the training protocol (** ***n*** **= 23)**

**Parameter**	**Before**	**After**
Body weight (kg)	76.9 ± 11.8	75.6 ± 12.3**
BMI (kg/m^2^)	30.8 ± 4.3	30.2 ± 4.5**
Waist circumference (cm)	93.4 ± 10.63	91.5 ± 10.7**
Hip circumference (cm)	106 ± 8.5	105.2 ± 8.6
Waist-hip ratio	0.87 ± 0.05	0.86 ± 0.06*
Total body lean mass (kg)	39.2 ± 5.3	39.6 ± 5.5*
Total body fat mass (kg)	33.7 ± 7.2	32.3 ± 7**
Truncal fat mass (kg)	17.9 ± 4.5	16.8 ± 4.5**
Arms fat mass (kg)	4 ± 0.9	3.8 ± 0.9*
Legs fat mass (kg)	11 ± 2.63	10.6 ± 2.63**
Resting heart rate (bpm)	66 ± 8.5	67.2 ± 9.8
Systolic blood pressure (mmHg)	123.4 ± 15.7	120.7 ± 14.8
Diastolic blood pressure (mmHg)	76.8 ± 11	75.6 ± 11.3
VO_2_max (ml · kg^−1^ · min^−1^)	32.1 ± 5.9	36.9 ± 5.6**
Total exercise test duration (min)	15.6 ± 2	17.3 ± 1.9**
Glucose (mg/dL)	110 ± 34.6	103.8 ± 26.3
Total cholesterol (mg/dL)	188.1 ± 46.3	204.4 ± 39.2
Triglycerides (mg/dL)	162.1 ± 108.6	124.1 ± 53.2
HDL (mg/dL)	34.1 ± 13.5	36.9 ± 7.8
Creatinine (mg/dL)	0.73 ± 0.1	0.67 ± 0.1

BMI, body mass index; VO_2_max, maximal oxygen uptake; HDL, high-density lipoprotein.

*P < 0.05 and **P < 0.001 after vs. before training.

### Energy intake characteristics

No differences were observed in total caloric intake and amount of carbohydrate, protein, and lipid consumed during the AT (Table 4).

**Table 4:** **Energy intake before and after the training protocol (** ***n*** **= 23)**

**Variables**	**Before**	**After**
Energy intake (kcal)	1,469.5 ± 394.1	1,379.3 ± 415.2
Carbohydrate (kcal)	801.9 ± 280.1	739.9 ± 263.7
Protein (kcal)	283.9 ± 127.8	261.7 ± 109.4
Lipid (kcal)	367.9 ± 163.3	378.6 ± 170.1

Values are expressed as mean ± SD.

### Oxidative stress markers

The levels of TBARS (*P* < 0.05) and AOPP (*P* < 0.001) decreased in women with MS after the AT protocol. Furthermore, an increase in T-SH levels (*P* < 0.001) was observed while the NOx levels were unchanged (*P* = 0.191) after the AT (Table 5).

**Table 5:** **Oxidative stress plasma parameters before and after the training protocol (** ***n*** **= 23)**

**Parameter**	**Before**	**After**
AOPP (μmol/L)	100.4 ± 34.5	69 ± 17.9**
NOx (μmol/L)	65.3 ± 40.7	53 ± 6.96
TBARS (μg/dL of MDA)	29.1 ± 10.5	24.3 ± 9.3*
T-SH (nmol/mg of GSH)	51.8 ± 12	69 ± 14.3**

AOPP, advanced oxidation protein products; NOx, nitrite and nitrate; TBARS, thiobarbituric acid-reactive substances; MDA, malondialdehyde; T-SH, total thiol content; GSH, glutathione.

*P < 0.05 and **P < 0.001 after vs. before training.

### Cytokine concentrations

**Figure 1:**
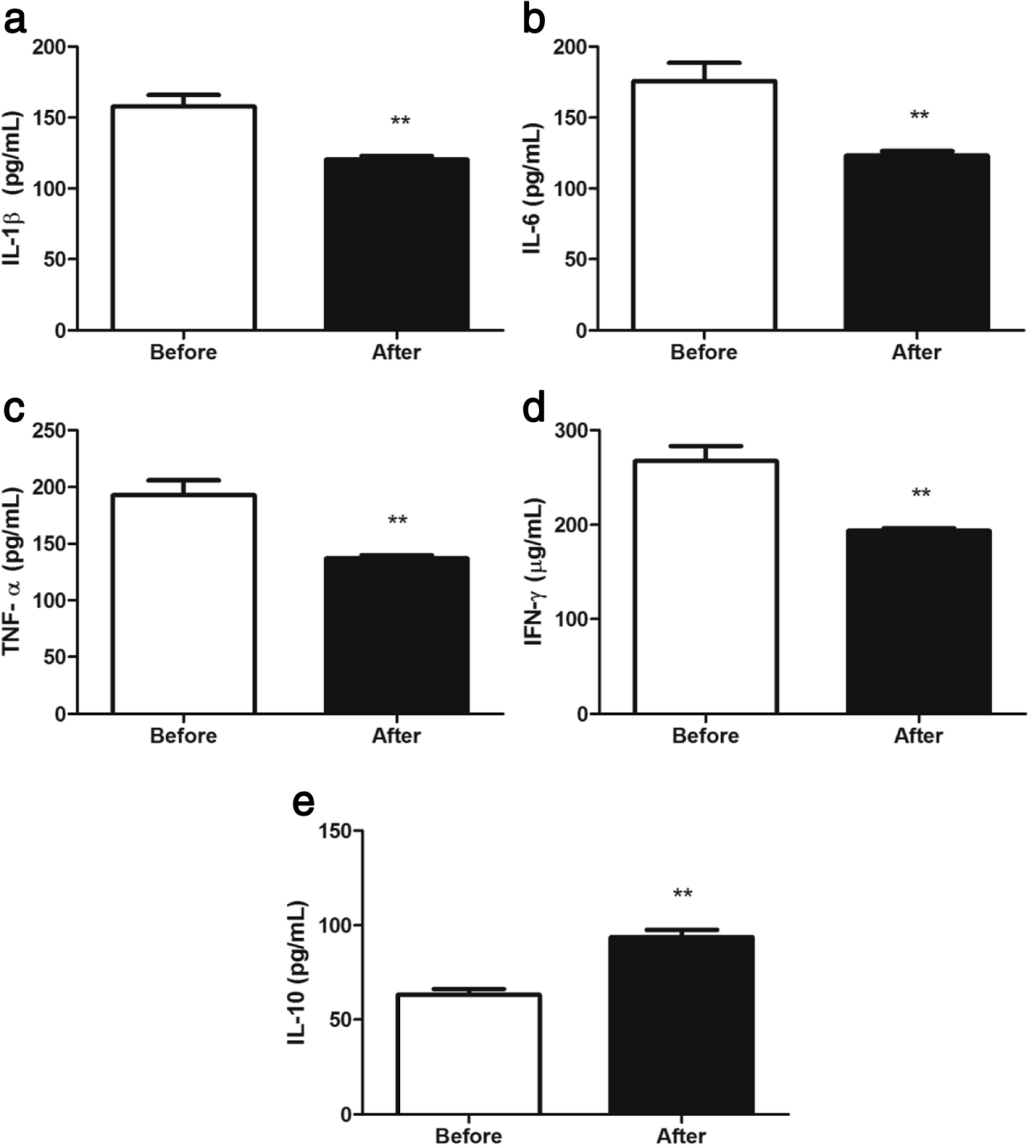
**Effects on systemic cytokine levels.** Aerobic training effects on interleukin-1 beta (IL-1β) (a), tumor necrosis factor alpha (TNF-α) **(b)**, interleukin-6 (IL-6) **(c)**, interferon-gamma (INF-γ) **(d)**, and interleukin-10 (IL-10) **(e)** levels. Data are expressed as means ± SD. *P < 0.05 and **P < 0.001 after vs. before training.

Twelve weeks of moderate-intensity AT resulted in a great effect on systemic cytokine levels (Figure 1). The serum levels of proinflammatory IL-1β (*P* < 0.001) (Figure 1a), IL-6 (*P* < 0.001) (Figure 1b), INF-γ (*P* < 0.001) (Figure 1c), and TNF-α (*P* < 0.001) (Figure 1d) decreased after the AT in women with MS. Besides, the concentration of the anti-inflammatory cytokine IL-10 (*P* < 0.001) was higher after the AT (Figure 1e).

### Changes in gene expression in PBMC

**Figure 2:**
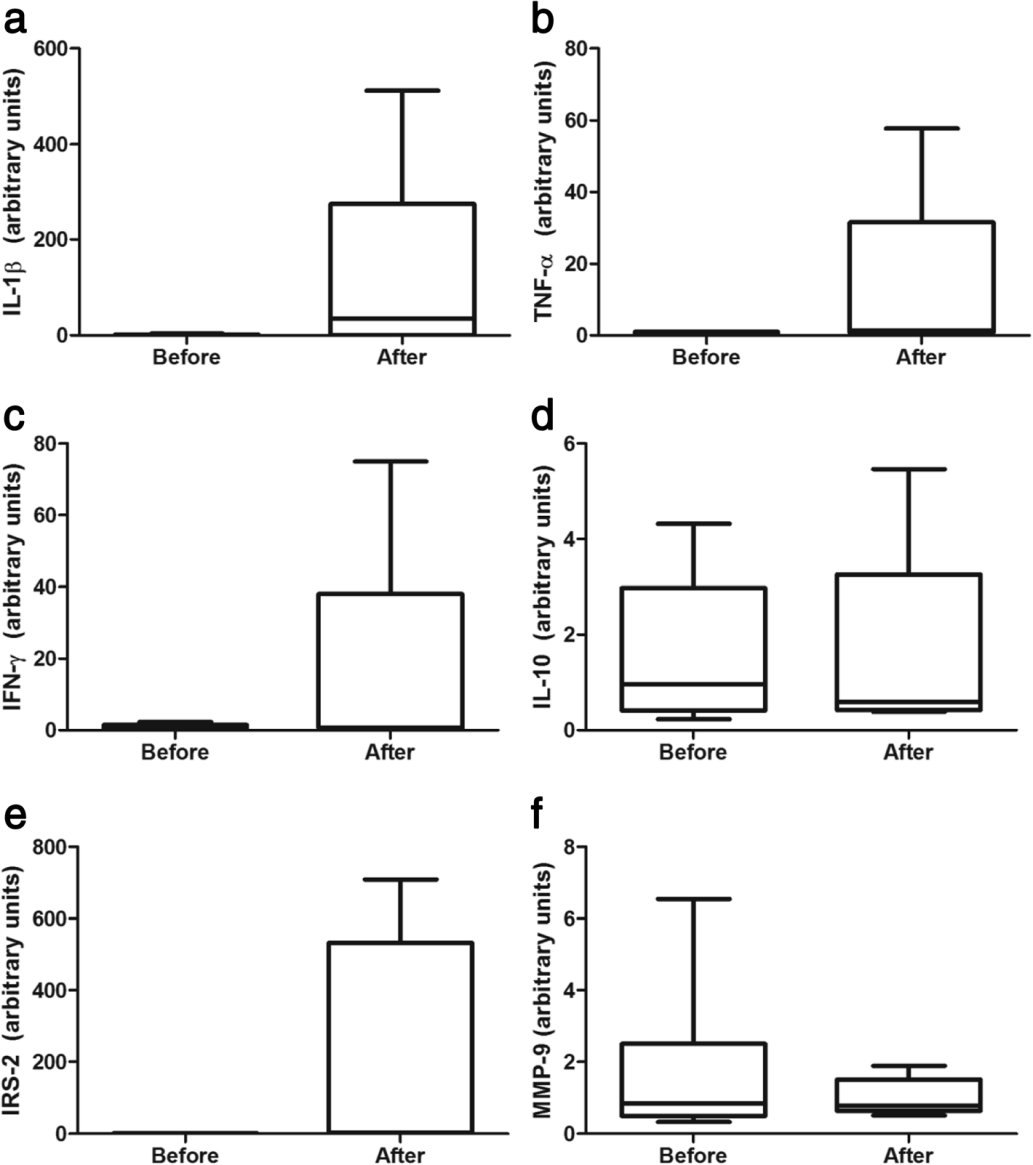
**IL-1β, TNF-α**, **IFN-γ, IL-10, IRS-2, and MMP-9** mRNA **expression in PBMC.** Box plots of aerobic training effects on interleukin-1 beta (IL-1β) **(a)**, tumor necrosis factor alpha (TNF-α) **(b)**, interferon-gamma (IFN)-γ **(c)**, interleukin-10 (IL-10) **(d)**, insulin receptor substrate 2 (IRS-2) **(e)**, and matrix metalloproteinase-9 (MMP-9) mRNA expression in PBMC of eight women with metabolic syndrome. Values are normalized to β-actin mRNA expression. Data are expressed as median, interquartile range, and whiskers extending to the 5th and 95th percentiles. *P < 0.05 and **P < 0.001 after vs. before training.

IL-1β, TNF-α, IFN-γ, IL-10, IRS-2, and MMP-9 mRNA expression in PBMC of a subset with eight women with MS was unchanged along the intervention (Figure 2).

## Discussion

The main findings of this study are that 12-week moderate-intensity aerobic training decreases oxidative stress and inflammatory parameters in women with metabolic syndrome, despite no changes in PBMC mRNA expression being observed. To our knowledge, this is the first study to investigate the effects of a moderate-intensity AT on PBMC mRNA expression of inflammatory and glucose metabolism genes using qPCR. It may also be assumed that the intensity and duration of the AT sessions were sufficient to induce metabolic and functional adaptations, considering that VO_2_max and duration of treadmill exercise test were increased. Moreover, it is noteworthy that despite the absence of changes in some MS criteria classification (such as TG, HDL, and blood pressure levels), 12 weeks of moderate-intensity AT led to improvements in the oxidative metabolism and inflammation profiles in this population. Despite no significant changes in total energy, carbohydrate, protein, and lipid intake observed in participants along the protocol, the low mean self-reported intake in a 3-day diet record (~1,430 kcal) should be linked to the recognized great underreporting in obese participants [25].

The AT here described induced total body and truncal fat mass decreases and body lean mass increases in MS women. Changes in total body and truncal fat are likely connected with the decreased cytokine concentrations, considering hypertrophied adipocytes are responsible for the cytokine release on obesity [5]. In this regard, the adipose tissue is considered not only such a fat depot but also an endocrine tissue linked to appetite modulation, insulin sensitivity, endocrine and reproductive systems, bone metabolism, inflammation, and immunity [26]. In fact, AT increases energy expenditure and conducts lipolysis of both subcutaneous and intramuscular fat stores [27] accumulated in individuals who ingest more energy than they need [28], such is the case of MS patients.

Elevated white adipose tissue, hyperglycemia, endothelial ROS production, and inadequate antioxidant defenses are connected to oxidative stress in obesity [8]. In the current study, the AT induced a decrease on oxidative damage (TBARS and AOPP) with a concomitant increase in antioxidant status (T-SH) in MS women, indicating oxidative balance. The long-term exercise-induced adaptations of oxidative stress are similar to the general principles of exercise training [11]. This suggests that the chronic exposure to prooxidant agents such as bouts of moderate aerobic exercises results in the upregulation of antioxidant defenses, providing a balance between the ROS-induced damage and the antioxidant systems [3,11]. At same time, the adipose tissue expression of NADPH oxidase, an important source of ROS production, was found downregulated by AT in rodents, with more remarkable effects in visceral than in subcutaneous white adipose tissue [29]. In conjunction, these factors led to oxidative damage repair, decreasing the risk of chronic diseases.

Nitrite and nitrate are products of nitric oxide (NO) and were unchanged in this study. The NO presents a dual physiological role, considering its beneficial and potent vasodilator function and its detrimental oxidative damage through peroxynitrite (ONOO^•-^) formation [30], reducing the bioavailability of NO for vasodilator, antihypertensive, and antiatherosclerotic effects. While a study involving a 12-week AT with elderly women found increased plasma levels of NOx [31], another encompassing 16-week AT with women at risk for hypertension showed no differences on this marker [32]. Moreover, it is likely that the lack of checking of the intake of food sources of NOx over 24 h prior to blood collections in our study had limited the comparison of this finding [31]. Another hypothesis is that exercise training induces the enlargement of conduit vessels, leading to the normalization of shear stress and decreased activation of endothelial nitric oxide synthase [33], probably linked to decreased plasma NO products.

This study found that a 12-week moderate-intensity AT reduced IL-1β, IL-6, TNF-α, and INF-γ serum levels, while IL-10 was increased. Some mechanisms have been proposed to explain how exercise training may reduce chronic low-grade inflammation in MS. It is known that as well as the adipose tissue, the working skeletal muscle is a potential source of cytokines. The IL-6 produced by myocytes through of AMP-activated protein kinase (AMPK) activation at sufficient AT intensities presents anti-inflammatory effects as opposed to IL-6 secreted by adipose tissue, promoting the release of IL-10 and interleukin-1 receptor antagonist (IL-1RA), with a concomitant inhibition of TNF-α production during the effort and some hours after the exercise sessions [6]. Another possible mechanism is the reduced expression of the toll-like receptors and nuclear transcription factor κB (NFκB) on monocytes and macrophages, probably linked to hormonal and heat shock protein levels, increased lipolysis, and reduced number of monocytes reported in some studies [5,28]. Moreover, recent evidences show that exercise training may increase angiogenesis and blood supply, thereby reducing hypoxia and the associated inflammation in adipose tissue [5]. Exercise training may also improve the capacity to regenerate endothelial cell after injury, increase laminar shear stress, and reduce the release of adhesion molecules, downregulating the leukocyte migration into the vessel wall and reducing local inflammation [5]. In summary, the mechanisms concerning lower systemic inflammation afforded by AT contribute to health achievement/improvement, represented by the protection against cardiovascular diseases among others [6] in MS women.

It is known that monocytes generate superoxide (O_2_^•^), hydrogen peroxide (H_2_O_2_), hydroxyl radical (OH^•^), and ONOO^•-^ and macrophages produce cytokines [8]. This leads to a repeated cycle of adipocyte-initiated macrophage recruitment and cytokine/ROS production by monocytes/macrophages [8]. In fact, PBMC are the primary cytokine secretors, and the infiltration/differentiation of these cells into macrophages in fat depots and/or endothelium represents a key role in the development of persistent tissue inflammation and atherosclerotic lesions [8,28,34]. Given the difficulties of hepatic, muscle, and adipose-associated immune cell collection, studies with PBMC may clarify mechanisms of immune dysfunction in MS. Concerning AT, evidences hypothesize that it may cause repeated short-lasting elevations in circulating levels of chemokines which downregulate the expression of their receptors on PBMC and restrict migration of these cells towards adipose tissue [28].

Previous studies concerning serum or plasma cytokine levels and their concentrations and/or mRNA expression in PBMC have evaluated the acute effects of exercise [13,16], the outcomes of a diet program [14], or the impact of caloric restriction combined with physical activity advising [15]. To our knowledge, this is the first study to investigate the effects of chronic exercise on inflammatory gene expression in PBMC. This study shows that a 12-week moderate-intensity AT may not change IL-1β, TNF-α, IFN-γ, IL-10, IRS-2, and MMP-9 mRNA expression levels in PBMC of MS women. However, it is important to mention that a reduced amount of PBMC samples was assayed, and thus this data interpretation must be taken carefully. Previous studies demonstrated that a 33-week caloric restriction in obese individuals with MS did not change IL-10 mRNA expression, while it decreased IL-1β, IL-1RA, TNF-α, and its receptors mRNA levels [14,35]. On the same line, a trial encompassing 12-week diet and regular walking advising, but not controlled, showed decreased mRNA expression for TNF-α and MMP-9 in obese women [15]. In common, these interventions provoked approximately 4.8% of weight loss [14,15,35], contrasting with our findings (~1.7%). Despite the statistical significance of body weight reduction found in our study, this decrease is not clinically relevant. Based on the aforementioned studies' findings [14,15,35], it could be hypothesized that major weight loss connected to exercise training is necessary to modulate inflammatory gene expression levels in PBMC. Moreover, it is noteworthy that although inflammatory gene expression in PBMC could reflect the production by these cells, it is not elucidated how it would interfere in the serum levels, considering mRNA expression do not directly reflect protein production and secretion [14,35]. It is also highlighted that the sample analyzed in this study presented a high standard deviation of mean in parameters regarding mRNA expression in PBMC. In this line, a more homogenous population could have led to different results in these variables.

## Conclusions

In conclusion, here, we describe that a 12-week moderate-intensity aerobic training induces positive effects on the oxidative stress and inflammatory modulation in women with metabolic syndrome. However, this AT does not appear to change mRNA expression of inflammatory and glucose metabolism parameters in PBMC. Moreover, despite study limitations such as the absence of a control group, cytokine protein expression, small sample considered for mRNA expression analysis, and the lack of control for the effect of estrogen in women with regular menstrual cycle, this study may serve as a basis to further randomize researches aimed at evaluating the effects of different exercise training programs on inflammatory parameters in immune cells of risk population. Furthermore, the effects of the same biomarkers after a period of detraining require clarification.
